# Telepharmacy during home isolation: drug-related problems and pharmaceutical care in COVID-19 patients receiving antiviral therapy in Thailand

**DOI:** 10.1186/s40545-023-00538-z

**Published:** 2023-02-24

**Authors:** Worapong Sungsana, Chotirat Nakaranurack, Benjabhorn Weeraphon, Watsa Charoenwaiyachet, Supparat Chanprasert, Pattama Torvorapanit, Wichai Santimaleeworagun, Opass Putcharoen

**Affiliations:** 1 Faculty of Pharmacy, Huachiew Chalermprakiet University, Samut Prakan, Thailand; 2 Department of Pharmacy Practice, Faculty of Pharmaceutical Sciences, Chulalongkorn University, Phayathai Road, Pathumwan, Bangkok, Thailand; 3 Faculty of Pharmaceutical Science, Burapha University, Chonburi, Thailand; 4 Department of Pharmacy, King Chulalongkorn Memorial Hospital, Thai Red Cross Society, Bangkok, Thailand; 5 Thai Red Cross Emerging Infectious Diseases Clinical Center, King Chulalongkorn Memorial Hospital, Bangkok, Thailand; 6 Infectious Disease Unit, Department of Medicine, Faculty of Medicine, Chulalongkorn University, Bangkok, Thailand; 7 Department of Pharmacy, Faculty of Pharmacy, Silpakorn University, Nakhon Pathom, Thailand

**Keywords:** Pharmacists, COVID-19, Coronavirus, Home isolation, Drug-related problems, Telepharmacy, Pharmaceutical care

## Abstract

**Background:**

Home isolation has been proposed for coronavirus disease 2019 (COVID-19) patients with mild symptoms to avoid hospital overcrowding. This study aimed to describe the drug-related problems (DRPs) and the pharmaceutical care of home-isolating COVID-19 patients in Thailand.

**Methods:**

Our cross-sectional study was undertaken from July 1 to September 30, 2021, at the King Chulalongkorn Memorial Hospital, Thailand. Patients who were ≥ 18 years old, were diagnosed with mild COVID-19 by real-time polymerase chain reaction (RT-PCR), and were able to isolate at home while receiving an antiviral agent and standard symptomatic treatment were enrolled. Infectious disease pharmacists provided a telepharmacy service on days 1 and 3 after the COVID-19 diagnosis.

**Results:**

A total of 197 patients met the study criteria. Their median age was 45 years, and their most common underlying disease was hypertension (44.29%). All patients exhibited excellent anti-COVID-19 drug adherence. We identified 125 DRPs, including adverse reactions (68%), and the unnecessary use of products (62.40%). Moreover, 91 patients (46.19%) reported the use of supplements or herbs, with vitamin C being the main supplement (37.36%). Pharmacists provided 36 recommendations and received 33 questions from COVID-19 patients.

**Conclusions:**

Our study demonstrates that telepharmacy is an essential service for detecting and preventing DRPs in home-isolating COVID-19 patients.

## Background

Coronavirus disease 2019 (COVID-19) is an infectious disease caused by the severe acute respiratory syndrome coronavirus-2 (SARS-CoV-2). The virus spreads rapidly from an infected person and has caused a global pandemic within a very short period of time [1].

The COVID-19 pandemic led to hospital overcrowding and hence shortage of beds and staff, which can affect the patients’ symptoms, clinical outcomes, and satisfaction [2]. Asymptomatic COVID-19 patients or patients with mild COVID-19 symptoms can be managed at home under proper medical guidance and monitoring. A previous study in China has shown that home isolation in COVID-19 patients with mild symptoms might be an effective way to relieve the strain of the medical and social resources during the COVID-19 pandemic and also achieve a satisfactory symptom improvement in these patients [3]. Telehealth has played a key role in providing remote health care for such patients during the COVID-19 pandemic [4–6], and it has been shown to improve patient satisfaction, disease monitoring, and medication compliance.

Factors associated with poor outcomes in home care patients are advanced age, multiple underlying diseases, and polypharmacy. Polypharmacy could lead to increased sensitivity to medication, drug–drug interactions, drug–disease interactions, and drug-induced toxicity [7]. Medication-related adverse events (AEs) have been associated with hospitalization in 2% of home care patients, whereas most events were considered to be ameliorable or preventable [8]. Several studies have found that pharmaceutical care can reduce drug-related problems (DRPs) and enhance clinical outcomes in both hospitalized patients and patients in home healthcare settings [7, 9–13]. During the COVID-19 pandemic, telepharmacy has become an important service through which healthcare authorities can provide pharmaceutical care to COVID-19 patients. Pharmacists can evaluate medication orders, provide counseling, provide drug information services, and advise for therapeutic drug monitoring (TDM). However, limited data exist regarding the role of telepharmacy and the DRPs of COVID-19 patients in home isolation in Thailand. This study aims to describe DRPs, pharmaceutical care, and drug information service of antiviral therapy-receiving, home-isolating COVID-19 patients in Thailand.

## Methods

This cross-sectional study was conducted at the King Chulalongkorn Memorial Hospital; a 1479-bed tertiary referral and teaching hospital in Bangkok, Thailand. The inclusion criteria were the following: (i) patients aged 18 years or older that were diagnosed with mild COVID-19 infection through a real-time polymerase chain reaction test; (ii) patients who were home-isolating between July 2021 and September 2021; and (iii) patients who received favipiravir for at least 5 days. Patients that did not appear for the planned follow-up on day 3 (through telepharmacy) were excluded. The Institutional Review Board (IRB) of the Faculty of Medicine of Chulalongkorn University has approved this study (IRB No. 0928/64).

The classification of COVID-19 disease severity modified from WHO [14] was performed as follows: for “mild” disease defined as symptomatic patients without evidence of viral pneumonia or hypoxia: (i) low risk of severe disease, asymptomatic or mild upper respiratory tract infection, and normal chest X-ray; (ii) high risk of severe disease with mild symptoms (including elderly, comorbidities such as lung disease, renal disease, cardiovascular disease, neurological disease, hypertension, diabetes mellitus, liver disease, immunocompromised host, and morbid obesity; body mass index, BMI > 30 kg/m^2^), upper respiratory tract infection, and a chest X-ray that was normal or showed minimal infiltration. “Moderate” disease was defined as symptomatic patients with clinical signs of pneumonia but no signs of severe pneumonia, including SpO_2_ ≥ 90% on room air, whereas “severe” disease was defined as symptomatic patients with clinical signs of pneumonia plus one of the following: respiratory rate > 30 breaths/min; severe respiratory distress; or SpO_2_ ≥ 90% on room air. Our study includes patients with mild disease with high risk of severe disease for home isolation 14 days, and treatment with favipiravir (standard dose of 200 mg, 8 tab po b.i.d, followed by 4 tab po b.i.d or high dose of 200 mg, 12 tab po b.i.d, followed by 5 tab po b.i.d for patients with BMI ≥ 30 kg/m^2^ or bodyweight > 90 kg) for at least 5 days, along with symptomatic treatment.

On the same day of their diagnosis, all patients received the COVID-19 treatment, a pulse oximeter, and a thermometer via the hospital logistics system. Healthcare providers followed up these patients to advise them regarding their clinical signs and symptoms, whereas the monitoring and handling of their problems were conducted via telephone or via the LINE application. The patients measured their body temperature and their finger oxygen saturation at least twice a day, and reported their symptoms to healthcare staff over the phone. Telepharmacy is a form of pharmaceutical care in which pharmacists and patients are not in the same place and can interact using information and communication technology (ICT) tools. Accordingly, pharmaceutical care in these settings defines as the provision of appropriate information on healthcare products, consultations, the supply of permanently available essential medicines for the management of the condition of the chronically ill, the identification and prevention of potential drug-related problems, and the follow-up of drug therapy [15]. In our hospital, infectious diseases pharmacists played an important role in the telepharmacy service by providing medication information and emphasizing drug adherence via telephone interview. They also followed up the patients to prevent and handle DRPs on days 1 and 3 after the diagnosis of the COVID-19 infection. Adverse reactions were also reported by the participating patients. If the symptoms began right after the drug was administered and no alternative causes that could solely have caused the events are identified, then these adverse reactions are recorded. Table 1 provides the classification of the DRPs employed by our study. Drug information service is an integral part of pharmaceutical care which provides medical information to patients, healthcare professionals, and other personnel including healthcare products information, treatment process, and others. Infectious disease pharmacists at our hospital answered and explained questions received from patients.

**Table 1:** The classification of DRPs, adapted from the Cipolle Strand Morley 2012 criteria [16]

Categories of DRPs	Description
Unnecessary use of products as part of the therapy	Products (such as drugs, herbs, or supplements) are unnecessarily used as part of the therapy, as the patient does not have a related clinical indication at the time
Need for additional drug therapy	Specific drug therapy is required to effectively treat or prevent a medical condition in the patient, but the patient does not receive this therapy at the time
Ineffective drug	The drug product fails to produce the desired response in the patient
Dosage too low	The dosage is too low to produce the desired response in the patient
Adverse reaction	The product is causing an adverse drug events in the patient
Dosage too high	The dosage is too high, resulting in undesirable effects being experienced by the patient
Nonadherence	The patient is not able or willing to take the drug therapy as intended
Drug interactions	The patient has a medical condition as a result of a drug–drug or a drug–food interaction

DRPs, drug-related problems

Data were collected from the e-PHIS-CUH program and the pharmacist’s recording form for COVID-19 patients; these data included demographic details, the patients’ COVID-19 vaccination profiles, the use of concomitant medications and supplements, recorded DRPs, the pharmacist’s intervention, and the questions received from patients. We have employed descriptive statistics for the analysis of the categorical variables (e.g., gender, comorbidities, concomitant drugs, drug adherence, DRPs, and the pharmacist’s intervention). Continuous variables are presented as mean ± standard deviation or median with IQR. All analyses were performed via STATA (version 14.0).

## Results

### Baseline characteristics of the study population

**Fig. 1:**
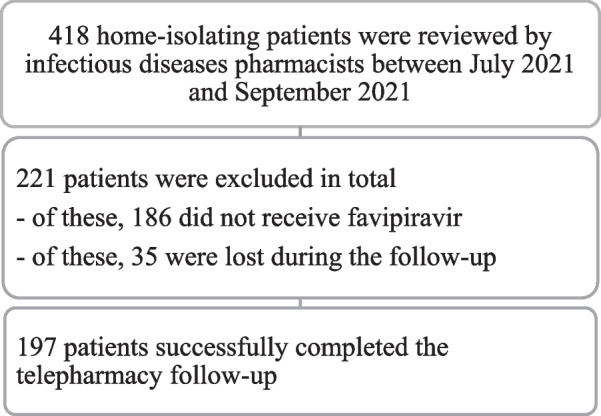
Flow diagram of the patient selection process followed in this study

A total of 197 patients met the inclusion criteria (Fig. 1). The median [interquartile range (IQR)] age was 45 (range: 33–55) years, and 51.26% of these patients were male. The most common underlying disease was hypertension, followed by dyslipidemia. Fifty-five (27.92%) patients were also receiving medication for a chronic illness, whereas vitamin C was the most consumed dietary supplement. Table 2 summarizes the demographic data of the selected COVID-19 patients.

**Table 2:** Demographic data of the selected home-isolating COVID-19 patients

Characteristic	*N* (%) (Total *N* = 197)
Gender	
Male	101 (51.26)
Age (years), median (IQR)	45 (33–55)
Underlying disease	70 (35.53)
Hypertension	31 (44.29)
Dyslipidemia	20 (28.57)
Diabetes mellitus	16 (22.86)
Liver disease	8 (11.43)
Obesity	7 (10.00)
Neurological disease	3 (4.29)
Cancer	2 (2.86)
Others	25 (35.71)
Concomitant of medication for chronic illness	55 (27.92)
Favipiravir dosage	
Standard dose	185 (93.91)
High dose	12 (6.09)
Post COVID-19 vaccination	121 (61.42)
1 dose	72 (59.50)
2 dose	48 (39.67)
3 dose	1 (0.83)
Concomitant of OTC, herb, and dietary supplement for COVID-19	91 (46.19)
Vitamin C	34 (37.36)
*Andrographis paniculata* extract capsule or tablets	29 (31.87)
*Boesenbergia rotunda* extract (total *n* = 25)	25 (27.47)
Capsule or tablet	11 (44.00)
Herbal drink	10 (40.00)
Inhalation	3 (12.00)
Crude herb	1 (4.00)
Ginger (total *n* = 22)	22 (24.18)
Herbal drink	21 (95.45)
Crude herb	1 (4.55)
Mixed herbs (total *n* = 15)	15 (16.48)
Capsule or tablet	2 (13.33)
Herbal drink	4 (26.67)
Inhalation	9 (60.00)
Others	29 (31.87)

IQR, interquartile range; OTC, over-the-counter medicine

### DRPs and pharmaceutical care

**Fig. 2:**
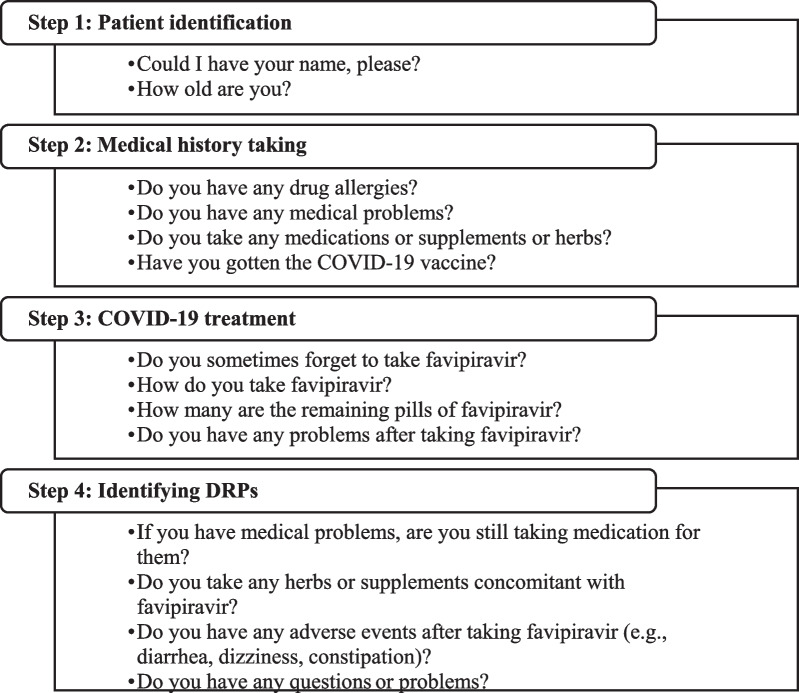
Flow diagram of telephone interview from the infectious diseases pharmacists. DRPs, drug-related problems

**Fig. 3:**
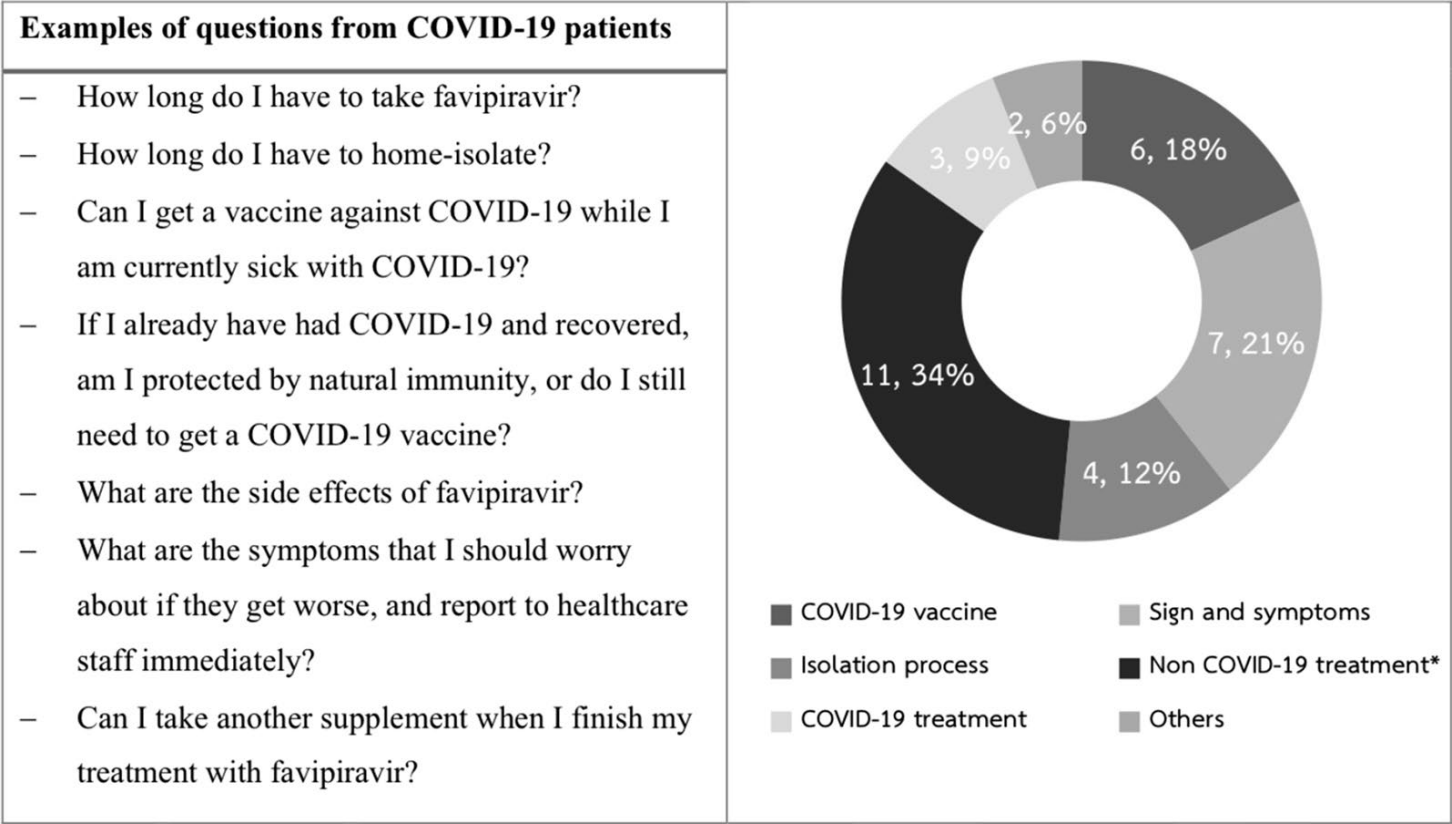
Examples of questions received from home-isolating COVID-19 patients (*n* = 33). *Non-COVID-19 treatment (including chronic medicine, over-the-counter medicine, and other supplements)

After completing the telepharmacy sessions (Fig. 2), we found that all patients have had good drug adherence for the COVID-19 treatment (100%). However, 11 patients (5.58%) were admitted to a hospital for further treatment due to a worsening of their symptoms during home isolation. Moreover, 125 patients (63.45%) reported DRPs, with adverse reactions as the most frequent, followed by unnecessary use of drugs or supplements as part of the therapy. Infectious disease pharmacists have advised all patients to stop using herbs or supplements, especially crude drugs or extraction products. Table 3 presents the identified DRPs and the pharmacists’ intervention-related data. Infectious disease pharmacists received 33 questions from home-isolating COVID-19 patients, and the leading question was about non-COVID-19-related treatments, followed by questions regarding the signs and symptoms of COVID-19. Figure 3 provides the examples and classification of these questions.

**Table 3:** DRPs and pharmacists’ interventions for home-isolating COVID-19 patients who received favipiravir as an antiviral treatment between July 2021 and September 2021

DRPs and pharmacists’ interventions	*N* (%) (Total *N* = 197)
DRPs	125 (63.45)
Adverse reactions	85 (68.00)
Diarrhea	32 (37.65)
Dizziness	15 (17.64)
Constipation	8 (9.41)
Nausea	7 (8.23)
Gastrointestinal discomfort	7 (8.23)
Somnolence	6 (7.06)
Headache	5 (5.88)
Vomiting	3 (3.53)
Dry throat/mouth	3 (3.53)
Chest tightness	2 (2.35)
Blurred vision	2 (2.35)
Faint	2 (2.35)
Rash	2 (2.35)
Fatigue	2 (2.35)
Sweating	1 (1.18)
Palpitation	1 (1.18)
Insomnia	1 (1.18)
Hair loss	1 (1.18)
Joint pain	1 (1.18)
Back pain	1 (1.18)
Unnecessary use of products as part of the therapy^a^	78 (62.40)
Nonadherence with medication for chronic illness	8 (6.40)
Needs for additional drug therapy	3 (2.40)
One patient had lung tuberculosis and needed additional therapy for the management of tuberculosis	
One patient was complaining about psychological symptoms (e.g., insomnia and anxiety)	
One patient had psoriasis and reported an exacerbation of the pathology’s skin-related symptomatology during home isolation	
Number of recommendations to the COVID-19 patients	36
Number of questions received from COVID-19 patients	33

DRPs, drug-related problems

^a^Products including *Andrographis paniculata* extraction products, *Boesenbergia rotunda* extraction products, ginger products, and other supplements

## Discussion

Our findings showed that during the COVID-19 pandemic, pharmacists play an essential role in drug-dosing special populations, delivering TDM and adverse drug reaction monitoring, delivering medication reconciliation, and preventing DRPs in hospitalized COVID-19 patients [4, 5, 17, 18]. Moreover, community pharmacists also play a major role by intervening on prescriptions for COVID-19 patients (by clarifying instructions for the use of medicine and by changing the medication order due to potential drug–drug interactions or contraindications) [6]. However, home isolation imposes restrictions on patient monitoring. The present study has shown that telepharmacy is an essential service for the detection of DRPs, and the delivery of counsel and a drug information service to home-isolating COVID-19 patients.

This study aimed to describe the DRPs in home-isolating COVID-19 patients receiving antiviral therapy, in Thailand. Generally, potentially inappropriate medications (PIMs) were additional drug therapy, wrong drug therapy, poor compliance, and adverse drug reactions (ADRs) [8]. The present study found that ADRs were a very common DRP for home-isolating COVID-19 patients, but none of these patients were affected severely enough from these AEs to be hospitalized. Our study also found that the suspected ADRs caused by favipiravir administration were similar to those reported in the World Health Organization (WHO) database, including nausea, vomiting, and diarrhea. Interestingly, a previous study has suggested that the most common AE of favipiravir is the elevation of hepatic enzymes [19].

They recommended that monitoring of the hepatic enzymes should be undertaken during treatment with favipiravir [19]. However, differentiating between the symptoms caused by favipiravir and those caused by the COVID-19 infection is difficult. Based on our findings, the incidence rate of ADRs should be used with caution.

In addition to the favipiravir treatment, the home-isolating COVID-19 patients sought herbs or supplements for the treatment of COVID-19; unsurprisingly, no sufficient evidence was found regarding the benefit and the safety of these substances in this clinical context.

In several studies, the *Andrographis paniculata* and *Boesenbergia rotunda* extracts have shown a potent inhibitory effect against SARS-CoV-2 [20, 21]. Moreover, Ayurvedic herbs (such as ginger) have been shown to significantly modulate the neutrophil function and may be useful in inhibiting the production of neutrophil extracellular traps and its consequences on platelet aggregation in COVID-19 [22]. However, the WHO has not recommended the use of any herbs or supplements for the management of COVID-19 [14]. The most common AEs recorded as a result of the consumption of the *Andrographis paniculata* extract were related to gastrointestinal, skin, and subcutaneous disorders [23]. There has also been a report on a hypersensitivity reaction among patients receiving an *Andrographis paniculata* extract that led to the patient’s hospitalization [24]. Healthcare professionals should be aware of serious AEs. Unnecessary drug therapy was common in our patients, especially in the form of the consumption of herbs along with favipiravir; this combination can enhance drug interactions and toxicities.

The pharmacists’ interventions in hospitalized COVID-19 patients are different from those afforded for home-isolating patients. In hospitalized patients, the dose adjustment of antithrombotic agents, antibiotics, or antiviral agents is commonly delivered by clinical pharmacists, especially in the case of patients with hepatic- or renal-impairments [4, 5, 18]. Our study showed that chronic disease-related problems (such as nonadherence or failure to receive medication for chronic diseases) are frequently encountered in home-isolating COVID-19 patients. The lockdown of different services decreased the follow-up and hospitalization of these patients, resulting in inadequate care for chronic conditions and inappropriate counseling, thereby leading to the adoption of irrational drug use [25]. Therefore, medication reconciliation (through telepharmacy) focusing on chronic disease-related medication, was necessary for home-isolating COVID-19 patients. Another important activity delivered through telepharmacy was the drug information service; the latter covered questions related to both COVID-19 and non-COVID-19 treatments. These data are particularly helpful for the improvement of the system.

In terms of vaccination, the study offered two doses of a COVID-19 vaccine to protect them against severe diseases and death [26]. More than one-half of the COVID-19 vaccinated patients in home isolation received one vaccine dose in our study. Thailand kickstarted the COVID-19 vaccination program in priority patients on February 2021 [27]; indeed, COVID-19 vaccination has been proven irreplaceable in preventing COVID-19 infection and severe diseases.

All home-isolating COVID-19 patients had displayed a good adherence to the prescribed COVID-19 treatment; a finding that is similar to that of a previous study [3]. Our home isolation system was supported by a multidisciplinary team, including physicians, nurses, and pharmacists. This system led to effective monitoring and ensured excellent patient compliance to the prescribed COVID-19 treatment.

This is the first study showing the DRPs in home-isolating COVID-19 patients.

We identified that the concomitant use of unnecessary products was common in this setting: an observation of particular concern when these products are used in parallel with antiviral agents. In this context, the pharmacists provided an effective drug information service, including the coverage of COVID-19- and non-COVID-19-related problems. Our study has some limitations. First, we provided a telepharmacy service on days 1 and 3 over the 14-day period of each patient’s home isolation because most patients received favipiravir for 5 days only; however, some patients might have experienced DRPs after the third-day follow-up. Second, most elderly patients were lost during the follow-up phase, and they were not able to provide accurate details regarding their chronic disease-related medications; thus, we might have gathered incomplete data regarding the ability of our telepharmacy service to deliver medication reconciliation and to identify or address DRPs. Finally, our study has only evaluated adult home-isolating COVID-19 patients; hence, we cannot possibly apply these data to pediatrics.

## Conclusion

Home isolation is a suitable option for the management of a COVID-19 infection with mild symptoms. Our study showed that telepharmacy with pharmaceutical care can play a key role in preventing and detecting DRPs in home-isolating COVID-19 patients. As part of such an approach, multidisciplinary collaboration is crucial and can ensure a successful outcome.

## Data Availability

The datasets that were used and analyzed during this study are not publicly available due to ethical considerations, as dictated by the Institutional Review Board approval.
